# The Impact of Bedrock Material Conditions on the Seismic Behavior of an Earth Dam Using Experimentally Derived Spatiotemporal Parameters for Spatially Varying Ground Motion

**DOI:** 10.3390/ma18133005

**Published:** 2025-06-25

**Authors:** Paweł Boroń, Joanna Maria Dulińska

**Affiliations:** Faculty of Civil Engineering, Cracow University of Technology, Warszawska 24, 31-155 Cracow, Poland; joanna.dulinska@pk.edu.pl

**Keywords:** earth dam, seismic analysis, geological conditions, multiple-support structure, spatial variability in ground motion, incoherence effect, wave passage effect

## Abstract

This study investigates the influence of bedrock material conditions on the seismic behavior of the Niedzica earth dam in southern Poland. It examines the dam’s dynamic response to a real seismic event—the 2004 Podhale earthquake—and evaluates how different foundation conditions affect structural performance under spatially varying ground motions. A spatially varying ground motion excitation model was developed, incorporating both wave coherence loss and wave passage effects. Seismic data was collected from three monitoring stations: two located in fractured bedrock beneath the dam and one installed in the surrounding intact Carpathian flysch. From these recordings, two key spatiotemporal parameters were experimentally determined: the seismic wave velocity and the spatial scale parameter (α), which reflects the degree of signal incoherence. For the fractured bedrock beneath the dam, the wave velocity was 2800 m/s and α = 0.43; for the undisturbed flysch, it was 3540 m/s and α = 0.82. A detailed 3D finite element model of the dam was developed in ABAQUS and subjected to time history analyses under three excitation scenarios: (1) uniform input, (2) non-uniform input with coherence loss, and (3) non-uniform input including both coherence loss and wave passage effects. The results show that the dam’s seismic response is highly sensitive to the choice of spatiotemporal parameters. Using generalized values from the flysch reduced predicted shear stresses by up to 16% compared to uniform excitation. However, when the precise parameters for the fractured bedrock were applied, the reductions increased to as much as 24%. This change in response is attributed to the higher incoherence of seismic waves in fractured material, which causes greater desynchronization of ground motion across the dam’s foundation. Even small-scale geological differences—when properly reflected in the spatiotemporal model—can significantly influence seismic safety evaluations of large-scale structures. Ultimately, shifting from regional to site-specific parameters enables a more realistic assessment of dynamic stress distribution.

## 1. Introduction

In the analysis of a structure’s dynamic response to seismic excitation, the time variability in ground motion is typically considered, while its spatial variability is often neglected. This simplification assumes that ground motion is identical at all support points—having the same amplitude and phase—an assumption known as uniform kinematic excitation. While this may be reasonable for small-scale structures, it becomes inadequate for large structures whose dimensions are comparable to the wavelengths of propagating seismic waves.

Experimental data from dense seismic arrays such as SMART-1, Lotung, Chiba, and Higashi-Matsuyama [1,2,3,4] confirm that ground motion amplitude and phase can vary significantly over short distances. These observations have led to the development of empirical and stochastic models that better represent the actual nature of earthquake excitation in so-called multiple-support structures. Modern ground motion models commonly incorporate three primary sources of spatial variability [5,6,7]: (1) wave passage effects—differences in the arrival times of seismic waves at various locations; (2) incoherence effects—the loss of coherence due to wave scattering and interference in the soil; and (3) local site effects—amplification or attenuation due to varying geotechnical conditions. Depending on the analysis, models of non-uniform excitation can range from simple (e.g., accounting only for wave passage) to complex (e.g., including all three effects).

Within the category of multiple-support structures, large-scale hydraulic structures such as dams are particularly sensitive to spatially varying earthquake ground motions (SVEGMs) due to their extensive spatial dimensions.

One of the earliest comprehensive seismic analyses of a dam was by Sodeify [8], who showed that wave propagation velocity in bedrock significantly affects dam response. Abouseeda and Dakoulas [9] analyzed a 100 m high earth dam, showing that spatial variability must be considered to avoid overestimating seismic demands. Papalou and Bielak [10] studied the La Villita dam, demonstrating that variations in soil layers and wave velocities produce differing abutment responses, consistent with real earthquake data. Chen and Harichandran [11] used a stochastic SVEGM model for the Santa Felicia dam, finding increased quasi-static effects in the drainage zone due to phase shifts and coherence loss. While most studies assumed perpendicular wave propagation, Magueri and Motta [12] analyzed parallel propagation and found reduced displacements under non-uniform excitation. Full-scale modeling by Sarmiento et al. [13] and Wang et al. [14] for the La Parota and Gongboxia dams confirmed the damping effect of wave passage. Bilotta et al. [15] observed asynchronous excitation in the El Infiernillo and Camastra dams via real-time monitoring. Adanur et al. [16] simulated the 1992 Erzincan earthquake at the Keban dam using different propagation velocities. Davoodi and Sadreddini [17] applied wave passage and incoherence to the 177 m high Masjed Soleyman dam, showing lower dynamic responses under SVEGM than uniform excitation. Isari et al. [18] introduced a symbolic regression-based method to model spatially varying inputs, validated with field data. Sadrolddini et al. [19] found that incoherent input signals reduce crest accelerations and displacements, warning of overestimation risks with uniform excitation. Davoodii et al. [20] compared three coherence models, showing that increased incoherency further lowers peak responses. Ghalyanchi et al. [21,22] studied eight dam geometries, finding that uniform excitation causes higher peak accelerations, while SVEGM may induce greater vertical displacements in smaller dams. They also found that wave angle and coherence can significantly increase shear stresses in critical zones. Nasiri et al. [23] used wavelet-based decomposition to produce an input closely matching recorded dam responses. A nonlinear 3D analysis by Mirzabozorg et al. [24] showed that SVEGM intensifies with stronger quakes, causing joint openings and stress concentrations. Dehghani et al. [25] found that incoherency-based inputs generally reduce stresses in arch dams. Yao et al. [26] showed that while the overall seismic response is lower under SVEGM for concrete-faced rockfill dams, tensile edge stresses on slabs may rise. Finally, Mostafaei et al. [27] presented a comparative study of pseudo-static and dynamic analyses applied to the stability assessment of rock wedges in an arch dam. The authors evaluated how different analytical approaches influence the prediction of the overall seismic behavior of the dam.

Overall, recent studies highlight the importance of incorporating non-uniform kinematic excitation in the seismic analysis of dams. Spatial variability in the seismic input may have a significant impact on the stress distribution within dam structures. Many of the reviewed studies confirm that uniform excitation typically overestimates seismic responses compared to more realistic non-uniform inputs. However, in some cases, non-uniform excitation can intensify localized effects (e.g., shear stresses at the base).

In this study, the dynamic response of the earth dam in Niedzica, Poland, to a real seismic event that occurred in the Podhale region in 2004 was investigated. To evaluate the dam’s seismic behavior, a spatially varying ground motion excitation model was applied, accounting for both wave coherence loss and wave passage effects.

By analyzing seismic data from monitoring stations located within and around the dam—specifically in the fractured bedrock beneath the structure and in the surrounding undisturbed Carpathian flysch formations—key spatiotemporal parameters were experimentally determined. These parameters include the wave propagation velocity and the space scale parameter, which quantifies the degree of incoherence between ground motion signals across the dam’s foundation. The parameters were obtained through the cross-correlation analysis of seismic recordings from stations placed at both monitoring sites, representing distinct geological conditions. Then, the dynamic response levels obtained using parameters for both sites were compared, and the potential error resulting from using general parameters adequate for the Carpathian flysch instead of the precise parameters specific to the bedrock beneath the dam was estimated.

The main objective of this study was to assess how bedrock material conditions influence the seismic response of an earth dam, with particular emphasis on the role of spatiotemporal parameters reflecting ground variability. Specifically, this study aimed to

1. Assess the error in dynamic stress estimation when using generalized spatiotemporal parameters derived from adjacent rock formations (e.g., Carpathian flysch), as opposed to experimentally derived parameters specific to the fractured bedrock directly beneath the dam;
2. Evaluate the consequences of neglecting the spatial variability in seismic excitation, a common simplification in analyses that assume uniform kinematic input.

To meet these objectives, the following tasks were undertaken:

- The experimental determination of the seismic wave velocity and the space scale parameter for two distinct geological conditions: (a) undisturbed Carpathian flysch surrounding the dam and (b) fractured bedrock directly beneath the dam structure.
- The numerical simulation of the dam’s dynamic behavior under three different kinematic excitation models: (a) uniform excitation (neglecting spatial variability), (b) non-uniform excitation incorporating only coherence loss, and (c) non-uniform excitation including both coherence loss and the wave passage effect.

The novelty of this research lies in the incorporation of experimentally derived spatiotemporal parameters, reflecting site-specific bedrock material conditions, into the seismic analysis of an earth dam. Unlike prior studies that rely on assumed or regionally averaged values, this approach uses in situ seismic measurements to accurately represent localized ground motion characteristics. By differentiating between fractured and intact bedrock, this study offers a more realistic and precise assessment of seismic demand and structural behavior. To the authors’ knowledge, the incorporation of measured spatiotemporal variability (especially the space scale parameter) in relation to specific bedrock conditions has not been previously addressed in the literature.

## 2. Materials and Methods

### 2.1. Theoretical Framework for the Dynamic Response of Multi-Support Structures Subjected to Spatially Varying Ground Motions

The dynamic behavior of multi-degree-of-freedom (MDOF) systems influenced by ground vibrations is governed by the following equations of motion [7,28]:

(1) $$\left[\begin{array}{cc} M_{ss} & M_{sg}\\ M_{gs} & M_{gg} \end{array}\right]\cdot \left(\begin{array}{c} {\ddot{x}}_{s}\\ {\ddot{x}}_{g} \end{array}\right)+\left[\begin{array}{cc} C_{ss} & C_{sg}\\ C_{gs} & C_{gg} \end{array}\right]\cdot \left(\begin{array}{c} {\dot{x}}_{s}\\ {\dot{x}}_{g} \end{array}\right)+\left[\begin{array}{cc} K_{ss} & K_{sg}\\ K_{gs} & K_{gg} \end{array}\right]\cdot \left(\begin{array}{c} x_{s}\\ x_{g} \end{array}\right)=\left(\begin{array}{c} 0\\ F_{g} \end{array}\right)$$

where

*s*, *g*—the degrees of freedom of the structure and the ground, respectively;
M, C, K—mass, damping, and stiffness matrices;
${\ddot{x}}_{s}$, ${\dot{x}}_{s}$, $x_{s}$—accelerations, velocities, and displacements for each DOF of the structure;
${\ddot{x}}_{g}$, ${\dot{x}}_{g}$, $x_{g}$—accelerations, velocities, and displacements for each DOF of the ground;
$F_{g}$—the reaction vector.

The total nodal displacement vector (*x*) is composed of dynamic ($x^{d})$ and quasi-static ($x^{q})$ components:

(2) $$x=\left(\begin{array}{c} x_{s}^{d}\\ 0 \end{array}\right)+\left(\begin{array}{c} x_{s}^{q}\\ x_{g}^{s} \end{array}\right)$$

where

$x_{s}^{d}$—the sub-vector of the dynamic nodal displacement of a structure;
$x_{s}^{q}$—the sub-vector of the quasi-static nodal displacement of a structure;
$x_{g}^{s}$—the sub-vector of the quasi-static nodal displacement of the ground.

In scenarios involving static structural behavior (i.e., when ground displacements are applied very slowly), the dynamic terms in Equations (1) and (2) can be disregarded, yielding the expression for quasi-static displacements:

(3) $$x_{s}^{q}=-K_{ss}^{-1}\cdot K_{sg}\cdot x_{g}$$

Substituting Equations (2) and (3) into Equation (1) results in the following:

(4) $$M_{ss}\cdot {\ddot{x}}_{s}^{d}+C_{ss}\cdot {\dot{x}}_{s}^{d}+K_{ss}\cdot x_{s}^{d}\;{=(M}_{ss}\cdot K_{ss}^{-1}\cdot K_{sg}-M_{sg})\cdot {\ddot{x}}_{g}-{(C}_{ss}\cdot K_{ss}^{-1}\cdot K_{sg}-C_{sg})\cdot {\dot{x}}_{g}$$

According to Eurocode 8 [29], the second term on the right-hand side of Equation (4) can be neglected due to its relatively minor influence, leading to a simplified expression for structural motion under kinematic excitation:

(5) $$M_{ss}\cdot {\ddot{x}}_{s}^{d}+C_{ss}\cdot {\dot{x}}_{s}^{d}+K_{ss}\cdot x_{s}^{d}={-(M}_{ss}\cdot K_{ss}^{-1}\cdot K_{sg}+M_{sg})\cdot {\ddot{x}}_{g}$$

Equations (2) and (5) make it evident that the total displacement of the structure is directly influenced by the ground acceleration vector $({\ddot{x}}_{g}$), which captures motion at the structural supports.

In dynamic analyses of structures with multiple supports, it is essential to consider ground motion at each support point. Since ground acceleration or velocity is typically recorded at a single location, a kinematic excitation model that accounts for spatial variability in ground motion must be introduced to simulate differential support motion.

The simplest model of ground motion that accounts for the non-uniformity of kinematic excitation is referred to in the literature as the “delayed excitation” model [7]. It assumes that points along the direction of wave propagation experience identical motion but with time delays determined by the wave velocity. However, this model does not incorporate signal incoherence effects resulting from wave reflection, interference, or scattering, nor does it consider amplitude variations due to changes in local soil properties. In this study, an advanced spatiotemporal model of seismic excitation was employed, which accounts for both the wave passage effect and the incoherence effect.

### 2.2. Conditional Random Field Simulation of Ground Motions for Multiple-Support Structures

This section presents a method for conditionally simulating the ground motion at multiple support points of a structure, using the spatiotemporal correlation function approach developed in [30,31,32]. The authors propose an efficient technique for generating spatially varying ground motions based on known data from recorded events. The method assumes linear behavior in both the soil and structure. To simplify computations, the spatial correlation function considers only the predominant frequency of the seismic event, which significantly reduces the complexity of the simulation process.

The simulation procedure described in [31] enables the generation of acceleration time histories at locations distant from the recording site. This is achieved by using actual seismic data along with a defined wave velocity and a spatial correlation function. The correlation function, expressing the relationship between the values of the ground motion field at two different points (*i* and *j*), is defined as

(6) $$K(D_{ij})=\sigma^{2}e^{\left(\frac{-\omega_{d}\cdot D_{ij}}{2\pi v\alpha }\right)}$$

where

*D_ij_*—the separation distance between two field points (*i*, *j*);
*ω_d_*—the predominant frequency of the shock;
*v*—the wave velocity;
*α*—the space scale parameter (*α* > 0), which controls the coherence drop and depends on local geological conditions;
*σ*—the standard deviation of the recorded shock.

For two spatial points, this correlation can also be expressed in matrix form:

(7) $$K=\left[\begin{array}{cc} K_{11} & K_{12}\\ K_{21} & K_{22} \end{array}\right]$$

To generate a random acceleration signal at point *j* ($x_{u}$), based on a known signal at point *i* ($x_{k}$), the following formula is applied:

(8) $$x_{u_{s}}=-a_{max}+2a_{max}\cdot r_{d},\;s=1,\cdots ,n$$

where

$a_{max}$—the maximum ground acceleration of the original record;
$r_{d\;}$—a uniform random variable from a range <0, 1>.

Assuming the ground motion follows a zero-mean Gaussian distribution, the conditional probability density function of the symmetrically truncated Gaussian distribution for the generated signal is

(9) $$f\left(x_{u}|x_{k}\right)={\left(1-l\right)}^{-\frac{1}{2}}{\left(detK_{c}\right)}^{-\frac{1}{2}}\cdot {\left(2\pi \right)}^{-\frac{1}{2}}\cdot exp(-\frac{1}{2\left(1-l\right)}{\left(x_{u}-m_{c}\right)}^{T}K_{c}^{-1}\left(x_{u}-m_{c}\right))$$

where

$x_{u}$—a vector of unknown values (generated signal).
$x_{k}$—a vector of known values (registered signal).
$K_{c}=K_{11}-{K_{12}K}_{22}^{-1}\cdot K_{21}$—the conditional covariance matrix of the random field.
$m_{c}={K_{12}K}_{22}^{-1}x_{k}$—a vector of conditional mean values.
l—the truncation parameter, dependent on the $\frac{a_{max}}{\sigma }$ ratio. When this ratio is 4.0 or greater, the truncation can be disregarded (l=0).

The generated vector $x_{u}$ is accepted only if it passes the von Neumann rejection criterion:

(10) $$R\leq f\left(x_{u}|x_{k}\right)$$

where

(11) $$R={\left(detK_{c}\right)}^{-\frac{1}{2}}\cdot {\left(2\pi \right)}^{-\frac{1}{2}}{\cdot r}_{d}$$

If the condition is not satisfied, a new $x_{u}$ is generated, and the evaluation is repeated using the above equations.

To apply this method, two key parameters must be specified to reflect local geotechnical conditions: the wave propagation velocity *v* and the space scale parameter α. The value of α depends on local geological and topographical conditions. Numerical examples from [30,31,32] suggest using values of α ranging from 0 to 100. This parameter controls the correlation between points of the field: higher α values indicate stronger spatial correlation. Conversely, in subsoil conditions where signal coherence is significantly reduced due to wave scattering, α tends to be lower. The scale parameter α should be obtained experimentally based on the data concerning the seismic wave propagation patterns in the ground.

### 2.3. Structural Morphology of the Analyzed Earth Dam

**Figure 1:**
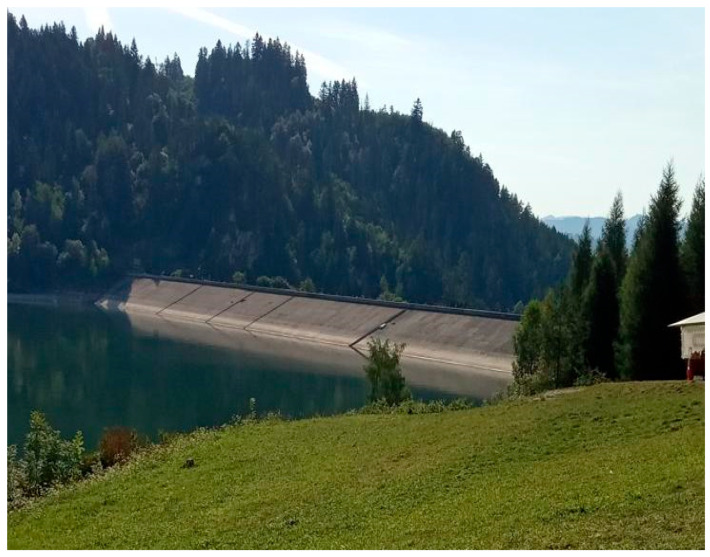
The Niedzica earth dam located on the Dunajec river [author’s photo].

The Niedzica earth dam (Figure 1) is located in the Podhale region, Southern Poland, on the Dunajec river. The longitudinal axis of the dam precisely aligns with the east–west direction. Detailed information on the geometry of the dam body, dam core, core protective layers, and drainage layer was obtained from the construction and post-construction technical documentation of the facility, as well as other works dedicated to the structural and constructional aspects of the dam [33,34,35].

**Figure 2:**
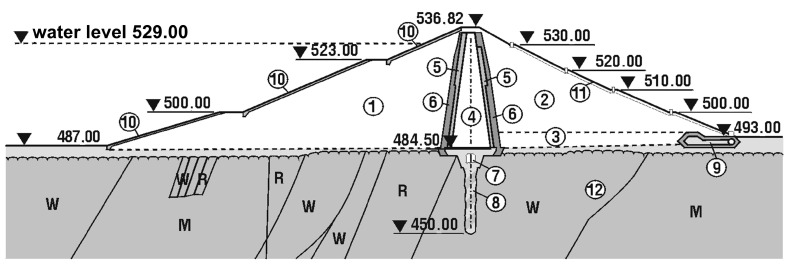
Typical cross-section of the Niedzica dam: (1) Coarse-grained dam body (upstream side) with 9 m wide berms on the slope. (2) Coarse-grained dam body (downstream side) with 3.50 m wide berms on the slope. (3) Drainage layer. (4) Clay core: width of the core crest—3.00 m; width of the core base—14.00 m; and slope inclination of the core—9:1. (5) Protective sand layer for the core, 1 m thick. (6) Protective gravel layer for the core, 3 m thick. (6) Inspection gallery in bedrock. (7) Cement curtain. (8) Dam drainage trench. (9) Concrete slabs—protection of the upstream slope. (10) Turf-protected downstream slope. (11) Bedrock: W—limestone; R—radiolarite; and M—marl.

The main dimensions of the structure are as follows: crest length—404.00 m; crest height—56.00 m; crest width—7.00 m; base width—256.00 m; core crest width—3.00 m; and core base width—14.00 m. A typical cross-section of the dam and its fundamental dimensions are presented in Figure 2. The dam body was constructed using aggregate and earth materials. The gravels and pebbles used during construction were sourced from local deposits located within the reservoir area. The sealing of the dam body was achieved using a central clay core. The body and the core form a continuous structure. Beneath the clay core, along the dam axis, there is an inspection and grouting gallery excavated in the bedrock, segmented approximately every 18 m. The upstream (water-side) slope of the dam is protected with concrete slabs measuring 3 m × 3 m × 30 cm, covered with two layers of bitumen felt. The downstream (air-side) slope is covered with turf. At the foot of the downstream slope, a drainage trench with inspection wells was constructed to collect rainwater.

### 2.4. Physical and Mechanical Properties of the Bedrock Material Under the Dam

To assess the seismic performance of the dam, it is necessary to determine the velocity of seismic wave propagation in the bedrock on which the dam is founded, as well as the coherence loss of the wave. These parameters depend on the physical and mechanical properties of the bedrock [36].

**Figure 3:**
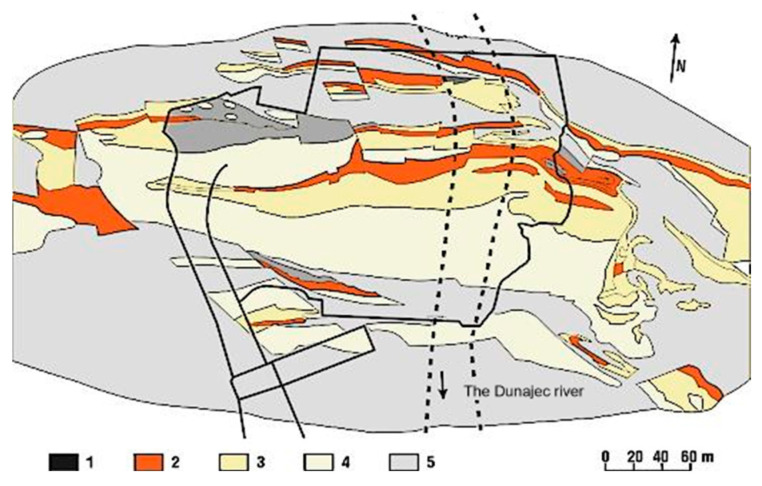
Geological map of the Niedzica dam area (based on [38]): 1—shales; 2—siliceous limestones; 3—radiolarites; 4—hornstones; and 5—marls.

The geological characteristics of the dam site’s bedrock were identified with particular precision before the dam’s construction. The Niedzica dam is located in the Pieniny Klippen Belt composed primarily of limestone formations. The Carpathian flysch surrounding the dam is a geologically stiff region, intact and unfractured. However, the foundation rock beneath the dam consists mainly of hornstone limestones, forming a band approximately 80 m wide along the dam. It is fractured and had been disturbed during dam construction. South of this limestone band are clay shales, marls, and marly limestones, while to the north, there are radiolarites, shales, and marls. On the west abutment, much of the rock mass is free from weathered cover, whereas on the east slope, the clay layer overlying the rock does not exceed 2 m in thickness. During the construction phase, the exposure of the foundation ground allowed for a detailed and accurate observation of the geological structure [37,38]. A geological map of the bedrock in the dam area is shown in Figure 3.

In the 1960s, detailed investigations into the mechanical properties of the bedrock were conducted, leading to a thorough understanding of the foundation conditions. The laboratory testing of rock samples enabled the determination of physical properties such as the specific weight, porosity, and water absorption. However, the primary focus was on determining mechanical parameters through field investigations, particularly using seismic methods [39]. These tests measured wave propagation velocities in the rock formations beneath the dam. The measured velocities of shear and compressional waves in the foundation made it possible to calculate the dynamic elastic modulus.

**Figure 4:**
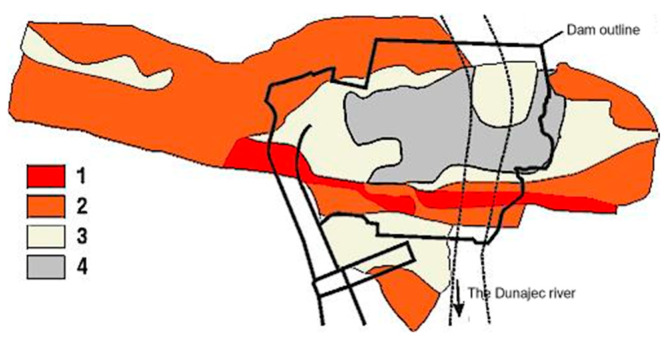
Distribution of elastic moduli of the dam’s bedrock—results from in situ tests (based on [38,39]): (1) E < 10 GPa, (2) 10 GPa < E < 20 GPa, (3) 20 GPa < E < 30 GPa, and (4) E > 30 GPa.

Figure 4 presents the distribution of the elastic moduli of the rock foundation beneath the dam. Table 1 summarizes the values of the dynamic elastic moduli and Poisson’s ratios of the dam bedrock material. Table 1 also includes theoretical shear and compressional wave velocities in the rock foundation, calculated using formulas for wave propagation in an elastic medium.

### 2.5. Assembly of the Numerical Model of the Dam

In the dynamic analyses of the Niedzica dam, a numerical model was used that incorporated all structural elements of the dam: the body, the core, the core’s protective layers, and the drainage layer. The process of the numerical model assembly was described in detail in [40,41]. It was assumed that the elastic moduli of the earth materials used in all structural elements of the dam depend on the mean effective stresses resulting from the dam’s self-weight and the water pressure in the reservoir, as well as on the degree of soil saturation. Similar assumptions regarding the distribution of the elastic modulus were adopted by the authors of studies [9,11,14,42,43].

In the numerical model of the dam, special attention was paid to the material properties of the earth dam. The dam material was assumed to behave as a Coulomb–Mohr elastic–plastic material. The parameters required to define the Coulomb–Mohr soil model were adopted based on the technical documentation of the structure [35]. On the downstream side, full soil saturation with water was assumed.

The dependence of the initial shear modulus of elasticity G_0_ (in the case of small-strain analysis) on the soil porosity index *e* and effective stresses was determined based on empirical formulas provided by Ishihara [44]. These formulas allow for the determination of the *G*_0_ modulus for various types of soil subjected to small deformations caused by dynamic effects.

The shear modulus *G*_0_ of the dam core, which is made of cohesive clay, was determined using the following formula [44]:

(12) $$G_{0}=3270\cdot \frac{{\left(2.97-e\right)}^{2}}{1+e}\cdot {\left(\sigma^{\prime }\right)}^{0.5}$$

where

*e*—porosity index;
$\sigma^{\prime }$—mean effective stress.

For the determination of the modulus *G*_0_ of the dam body, drainage, and the protective layers of the core, the following formula was used [44]:

(13) $$G_{0}=7230\cdot \frac{{\left(2.97-e\right)}^{2}}{1+e}\cdot {\left(\sigma^{\prime }\right)}^{0.38}$$

The elasticity modulus *E*, corresponding to the shear modulus *G*_0_, was determined using the following formula [45]:

(14) $$E=G_{0}\cdot 2(1+v)$$

Additionally, full water saturation was assumed for the downstream slope up to the water impoundment level, as well as for the drainage layer. In the fully saturated zones, the elastic modulus was determined based on the following relationship [45]:

(15) $$E_{und}=\frac{3\cdot E}{2\cdot (1+v)}$$

where

$E_{und}$—the elastic modulus and Poisson’s ratio of the dry material;
ν—Poisson’s ratio.

**Figure 5:**
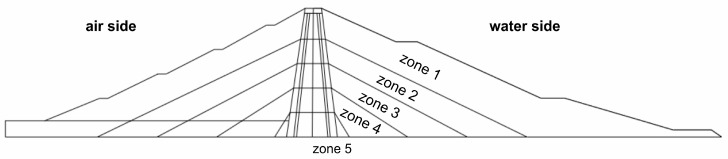
Division of the Czorsztyn–Niedzica earth dam into material zones.

The model was divided into 5 material zones, each with constant elastic moduli determined based on the previously presented Formulas (12)–(15). The division of the typical cross-section into material zones is shown in Figure 5.

Table 2 summarizes the values of average elastic moduli adopted in the individual zones of the dam’s computational model. A similar zoning model with different physico-mechanical parameters was proposed by Abuseeda and Dakoulas [46].

The numerical model of the dam takes into account the interaction of water using the formula proposed by Westergaard [47], which defines the mass of “bound water” associated with the dam structure and “co-vibrating” with the structure during dynamic loadings. Westergaard’s formula is commonly used in computational models of dams. This formula accounts only for the mass of “bound water” and does not consider the dynamic interaction of the fluid medium. For the vibration amplitude levels analyzed in this study, which occur during seismic shock, neglecting the dynamic effects of water is justified. A similar approach to modeling the interaction of water mass using Westergaard’s formula was also adopted by other authors, including Ghrib and Tinawi [48], who analyzed the dynamic response of the Koyna Dam to an actual seismic shock with a magnitude of 6.5 on the Richter scale. The results of the dynamic response calculations of the dam to the seismic shock, obtained using this relatively simple, classical method of considering the interaction of water in the reservoir, are very similar to those obtained using alternative methods for considering hydrodynamic pressure [49,50]. Although more advanced fluid–structure interaction methods can offer increased accuracy in certain cases, for the purposes of this study, Westergaard’s approach provides a reliable and computationally efficient approximation of the hydrodynamic effects.

**Figure 6:**
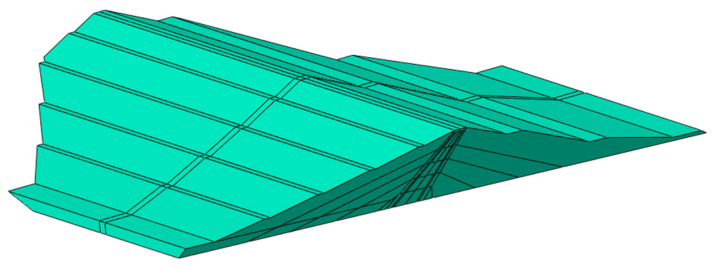
The three-dimensional numerical model of the Niedzica dam, showing the structural elements in the cross-section: the body, core, protective layers of the core, drainage zone, and division into material zones.

The dynamic calculations of the dam’s response to kinematic excitations were performed using the ABAQUS software [51], which enabled detailed time history analyses under the ground motion scenario. However, due to the geometric complexity of the dam—particularly its zoned structure and broad base width—the modeling process was supported by AutoCAD 2024, which facilitated the precise definition of the dam’s geometry. Tetrahedral finite elements from the program’s element library were used, which allowed for a more accurate representation of the complex geometry of the structure compared to rectangular elements. The number of elements was approximately 20,000. Due to the fact that the earth dam is founded on a thin layer of native soil, not exceeding 6 m in thickness, this layer was included in the model and covered with the finite element mesh. Figure 6 shows the three-dimensional numerical model of the structure, with the structural elements visible in the cross-section: the dam body, core, and protective layers of the core.

### 2.6. Experimental Setup in the Dam Area and Theoretical Background for Apparent Wave Velocity Estimation

**Figure 7:**
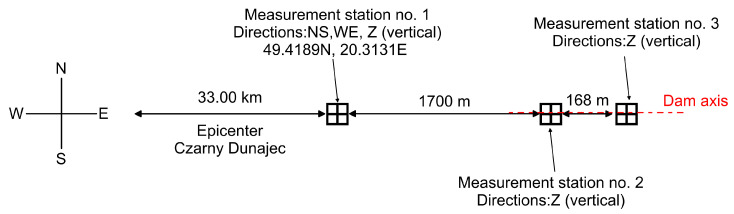
The arrangement of measuring equipment of the seismic stations in the area surrounding the Niedzica dam.

Since 1997, the area surrounding the Niedzica dam has been continuously monitored to assess the effects of local Carpathian seismic activity. Figure 7 illustrates the arrangement of measuring stations near the dam. The primary station—Station 1—records ground vibration velocities in three directions: horizontal (NS and WE) and vertical (Z). It is located in a bunker approximately 1700 m west of the dam and is equipped with SM-3-type seismometers. Additionally, Stations 2 and 3 monitor the vertical (Z) vibration velocities of the dam using two seismometers installed in the inspection gallery (see Figure 2), which is drilled into the dam’s bedrock. All stations record real-time velocity data with an accuracy of 1 ms. This experimental setup enables the recording of time histories, which can be used to determine the apparent wave velocity and the coherence parameter.

The apparent wave velocity *v* represents the speed at which a wave propagates beneath a structure with multiple supports. As the wave travels, it causes uneven displacements among the supports, leading to additional structural stresses. This apparent velocity tends to be higher when the underlying soil is stiffer.

To estimate the apparent wave velocity, one can use the cross-correlation function R(τ) of time-domain signals *x*(*t*) and *y*(*t*), recorded at two separate locations aligned along the wave’s direction of travel [52,53]:

(16) $$R(\tau )=\underset{\tau }{max}\int_{-T/2}^{T/2}x(t)\cdot y(t+\tau )dt$$

This function reaches its peak at a specific time shift *τ*, known as the time delay—the duration it takes for the wave to travel from one point to the other. With the known distance *dL* between the measurement points and the identified time delay, the apparent wave velocity can be calculated as

(17) $$v=\frac{dL}{\tau }$$

Since the time delay *τ* corresponds to the actual wave travel time, the signals *x(t)* and *y* (*t + τ*)align most closely in the time domain. Importantly, the apparent wave velocity reflects the average speed of the full wave signal, which is a combination of components at various frequencies. To assess the velocity of a single-frequency component, the signals must first be bandpass-filtered at that frequency before computing their cross-correlation to determine the corresponding time delay.

### 2.7. Characteristics of a Natural Seismic Shock Used for Dynamic Analyses

In November and December 2004, a series of seismic shocks occurred in the Podhale region of southern Poland, affecting the area around the Niedzica dam. The strongest quake took place on 30 November 2004, reaching a magnitude of 4.7 on the Richter scale, according to the European–Mediterranean Seismological Centre and Institute of Geophysics, Polish Academy of Sciences [54]. The seismic activity exhibited a swarm-like pattern—typical for the Western Carpathians—with several aftershocks occurring over a short period. The most significant aftershock, with a magnitude of 3.6, was recorded on 2 December 2004. The epicenters of both quakes were located in the village of Czarny Dunajec, approximately 33 km west of the Niedzica dam (see Figure 7). The 2004 Podhale seismic events were among the strongest ever recorded in Poland and caused damage to infrastructure in the region [55].

The main shock on November 30 revealed limitations in the monitoring equipment at the Niedzica seismological stations. Although the quake was recorded, the amplitude of the ground motion exceeded the instruments’ measurement range, which had been optimized for detecting weaker seismic events. Consequently, the data from that event could not be used in calculations related to the dam’s structural response. However, recordings from the December 2 aftershock proved valuable.

**Figure 8:**
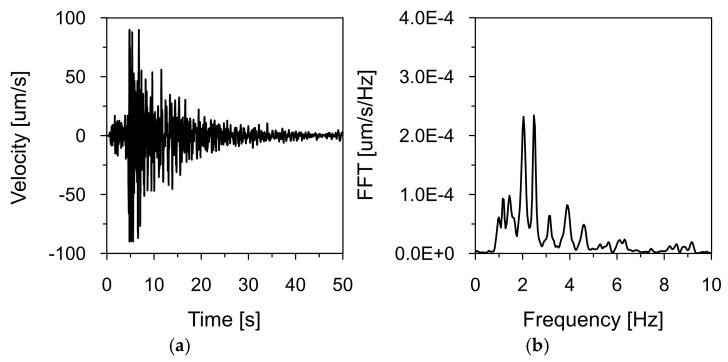
Ground vibration velocity in the horizontal direction (NS) during the seismic tremor on 2 December 2004, recorded at Station 1: (**a**) time history and (**b**) frequency spectrum.

**Figure 9:**
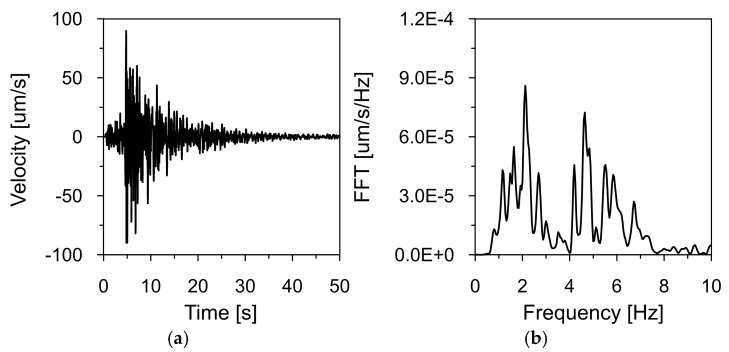
Ground vibration velocity in the horizontal direction (WE) during the seismic tremor on 2 December 2004, recorded at Station 1: (**a**) time history and (**b**) frequency spectrum.

**Figure 10:**
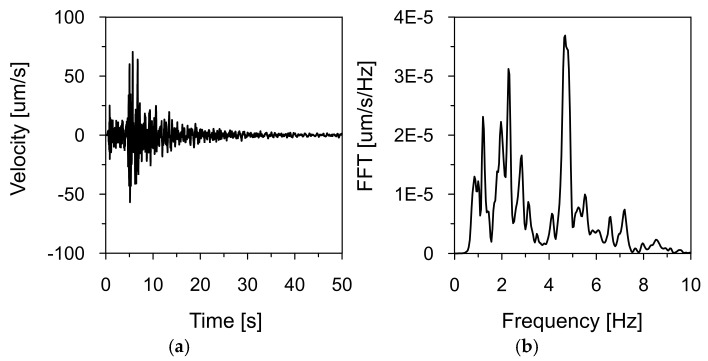
Ground vibration velocity in the vertical direction (Z) during the seismic tremor on 2 December 2004, recorded at Station 1: (**a**) time history and (**b**) frequency spectrum.

Figure 8, Figure 9 and Figure 10 show the time histories of the ground vibration velocities in the horizontal directions (NS and WE) and the vertical direction (Z), along with their corresponding frequency spectra, for the 2 December 2004 tremor recorded at Station 1, located 1700 m west of the dam (see Figure 7). These recordings were used as input data for kinematic excitation in the analysis of the dam’s dynamic response.

The tremor lasted approximately 30 s, with the intense shaking phase lasting 15 s. Frequency spectrum analysis revealed dominant vibration frequencies ranging from 1 to 3 Hz, with peak values near 2.31 Hz. Additionally, high amplitude values were observed in the 4–5 Hz range. These frequency bands are consistent with typical distributions observed for earthquakes in the Western Carpathians region.

**Figure 11:**
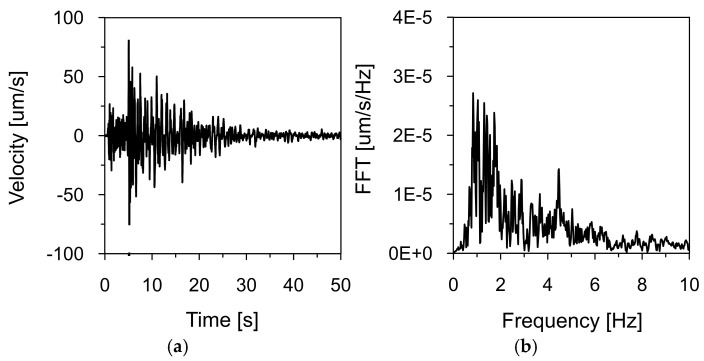
Ground vibration velocity in the vertical direction (Z) during the seismic tremor on 2 December 2004, recorded at Station 2: (**a**) time history and (**b**) frequency spectrum.

**Figure 12:**
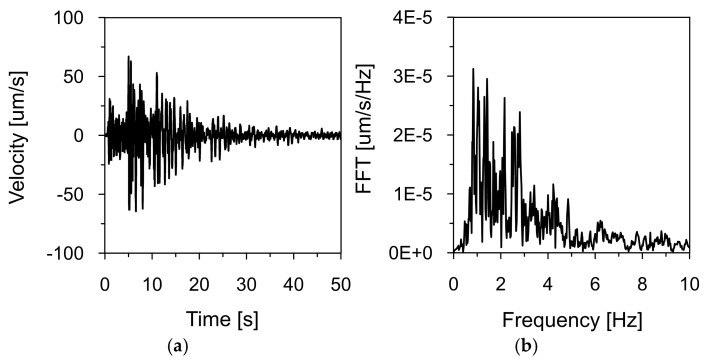
Ground vibration velocity in the vertical direction (Z) during the seismic tremor on 2 December 2004, recorded at Station 3: (**a**) time history and (**b**) frequency spectrum.

Figure 11 and Figure 12 present the time histories of the ground vibration velocities in the vertical direction (Z), along with their corresponding frequency spectra, recorded at Stations 2 and 3, located within the inspection gallery drilled into the dam’s bedrock (see Figure 2). As in previous recordings, frequency spectrum analysis indicated that the dominant vibration frequencies ranged from 1 to 2 Hz.

A summary of the main parameters of the 2 December 2004 Podhale aftershock is provided in Table 3.

### 2.8. Limitations of This Study

Having considered the above-mentioned materials and methods, this study has several limitations that should be acknowledged. Firstly, the analysis was based on a single real earthquake event—the 2004 Podhale earthquake—which, despite providing high-quality and spatially distributed ground motion records, may not fully represent the range of potential design-level seismic scenarios. The adopted approach allows for the accurate determination of spatiotemporal ground motion parameters at the observed amplitude level; however, it may not fully capture the variability and intensity associated with stronger seismic events that could affect the structure. Secondly, the dam and foundation materials were modeled assuming linear elastic behavior, which neglects potential nonlinear effects that may become significant during intense shaking. Thirdly, this study did not incorporate full fluid–structure interaction, which could influence the dynamic response of the dam, especially in the presence of a large reservoir. Addressing these limitations in future work—by including multiple earthquake scenarios, nonlinear material behavior, and hydrodynamic interactions—will enhance the robustness and applicability of the seismic safety assessment.

## 3. Results and Discussion

### 3.1. Determination of Wave Velocity and Space Scale Parameter for Different Bedrock Material Conditions

**Figure 13:**
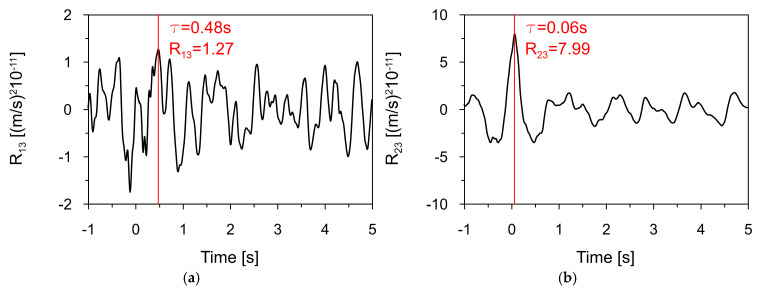
The cross-correlation functions between signals at (**a**) Station 1, located 1700 m from the dam, and Station 3, located directly beneath the dam, and between signals at (**b**) Stations 2 and 3, both situated within the inspection gallery beneath the dam and separated by 168 m.

The first step of this study involved comparing time series recorded during the earthquake (see Figure 10, Figure 11 and Figure 12) to identify similarities and time shifts between them. This analysis was conducted using the cross-correlation method (see Section 2.6). To investigate the influence of the bedrock material on the space scale parameter and seismic wave velocity, two cross-correlation functions were computed: (1) between signals recorded at Station 1, located 1700 m from the dam, and Station 3, located directly beneath the dam, and (2) between signals recorded at Stations 2 and 3, both situated within the inspection gallery beneath the dam and separated by 168 m (see Figure 7). The resulting cross-correlation functions are shown in Figure 13a and Figure 13b, respectively. The peak of each function indicated the time delay in the arrival of the seismic wave between the two sensors.

The cross-correlation between signals from Stations 1 and 3 (see Figure 13a) revealed a wave time shift of 0.48 s over a distance of 1700 m, which includes a section of intact, unfractured material of the Carpathian flysch bedrock. In contrast, the cross-correlation of signals from Stations 2 and 3 (see Figure 13b) beneath the dam revealed a precise delay of 0.06 s over a distance of 168 m. This shorter delay reflects wave propagation through fractured bedrock, which had been disturbed during dam construction.

With these time delays determined, the apparent seismic wave velocities were estimated by dividing the distance between each sensor pair by the corresponding time delay. The results provided apparent wave velocities in the different bedrock materials. The wave velocity in the intact Carpathian flysch bedrock was calculated to be 3540 m/s, while in the fractured rock beneath the dam, it was 2800 m/s. These results clearly demonstrate the significant impact of bedrock conditions on the seismic wave velocity.

The maximum value of the cross-correlation function between two signals reflects the degree of similarity or coherence between them at their point of best alignment. A higher peak value indicates that the signals are more similar in shape and content, suggesting stronger correlation. Based on Equation (6), the space scale parameter α was determined for different bedrock materials.

In calculating α for the intact Carpathian flysch, the following data were used:

*D*_12_ = 1700 m (separation distance between Stations 1 and 2);
*ω_d_* = 2.31 Hz (predominant frequency of the shock, see Figure 6);
*v =* 3540 m/s (seismic wave velocity in the intact Carpathian flysch bedrock);
*σ =* 6.19 × 10^−6^ (standard deviation of the shock recorded at Station 1);
K (1700 m) = 1.27 (the peak cross-correlation value between signals at Stations 1 and 3).

In calculating α for the fractured rock beneath the dam, the following data were used:

*D*_23_ = 168 m (separation distance between Stations 2 and 3);
*ω_d_* = 2.31 Hz (predominant frequency of the shock);
*v =* 2800 m/s (seismic wave velocity in the fractured rock beneath the dam);
*σ =* 6.19 × 10^−6^ (standard deviation of the shock recorded at Station 2);
K (168 m) = 7.99 (the peak cross-correlation value between signals at Stations 2 and 3).

Based on Equation (6), the space scale parameters of 0.82 and 0.43 were calculated for the intact Carpathian flysch bedrock and for the fractured rock beneath the dam, respectively.

### 3.2. Dependence of the Dam’s Seismic Performance on the Space Scale Parameters Determined for Different Bedrock Materials

The objective of this study was to elucidate the dependence of the dam’s seismic performance on space scale parameters associated with varying material properties of the underlying bedrock. To achieve this aim, seismic response analyses were conducted employing both uniform and non-uniform seismic excitation models, incorporating the effects of wave incoherence. Two distinct space scale parameters were utilized to assess the dam’s seismic behavior. The first parameter *α* = 0.82 represents an approximate value corresponding to the undisturbed Carpathian flysch in the vicinity of the dam. The second parameter *α* = 0.43 represents a precise value for the fractured bedrock beneath the dam.

The dynamic response of the dam to seismic shock was calculated using the time history analysis (THA) method, implemented in the Abaqus software. This approach involves the direct integration of the equations of motion at each time step and is known for its high accuracy. The THA simulations incorporated Rayleigh damping, based on mass and stiffness proportionality [54].

To compare the level of seismic response of the dam with different space scale parameters, the maximum shear stress (Tresca stress) was analyzed at selected points of the dam. The maximum shear stress in soil is a fundamental concept in geotechnical engineering, playing a critical role in the design and analysis of earth structures. It refers to the greatest shear stress that a soil can sustain before failure occurs, typically manifested through plastic deformation, sliding, or structural collapse. The maximum shear stress τ is determined using the following equation:

(18) $$\tau =\left|\frac{\sigma^{max}-\sigma^{min}}{2}\right|$$

where *σ^max^* and *σ^min^* are the maximum and minimum principal stresses, respectively.

**Figure 14:**
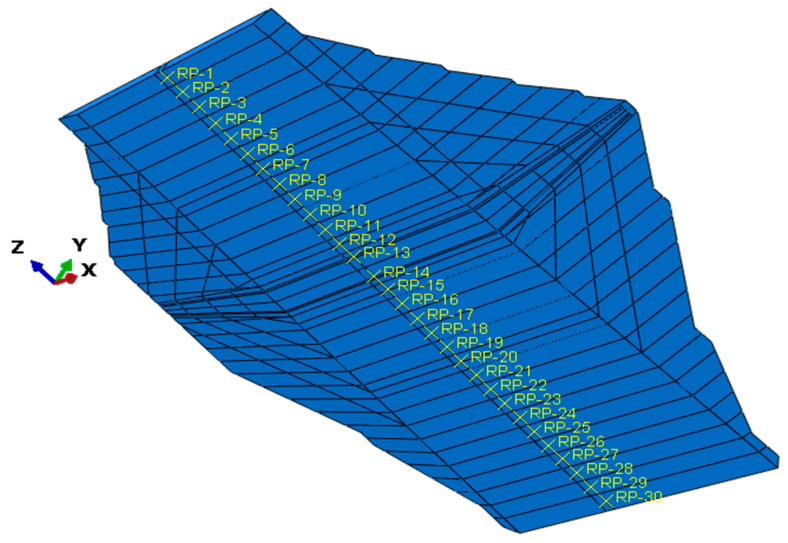
The division of the dam base into 30 partitions (RP-1 to RP-30) for the incorporation of wave propagation beneath the dam.

In order to input data related to the wave propagation beneath the dam, the base surface of the dam was divided into 30 partitions, spaced approximately every 10 m (Figure 14). Each partition was assigned seismic excitation data reflecting the wave passage effect and incoherence effect.

**Figure 15:**
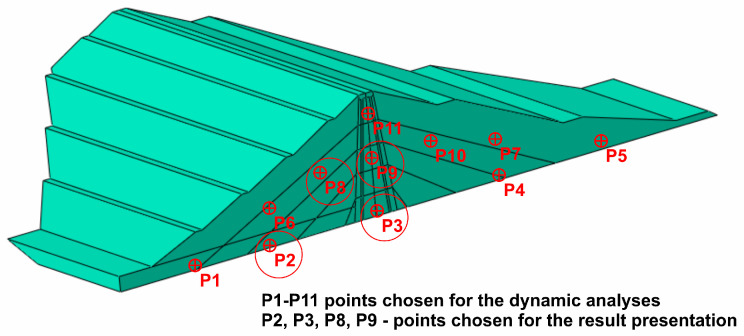
Points chosen for the seismic analyses as well as for the result presentation.

The points chosen for the analysis as well as those selected to present the results are shown in Figure 15. The representative points chosen for the result presentation are as follows: P2—located close to the dam base within the drainage layer; P3—located close to the dam base in the clay core; P8—located in the middle of the coarse-grained dam body; and P9—located in the middle of the dam clay core.

**Figure 16:**
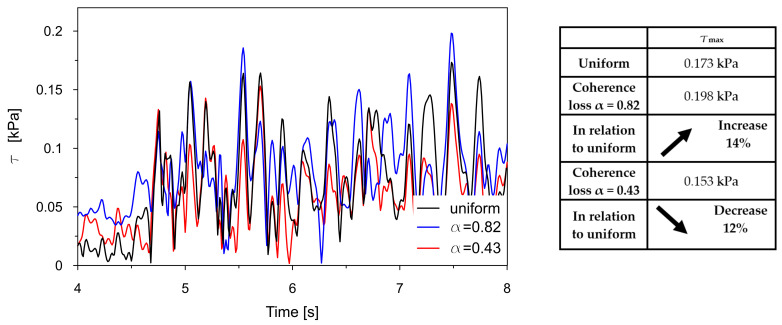
Comparison of the Tresca stresses at point P2, located near the dam base within the drainage layer, for the uniform excitation (black line) and the non-uniform excitation with the approximate space scale parameter *α* = 0.82 (blue line) and the precise one *α* = 0.43 (red line).

**Figure 17:**
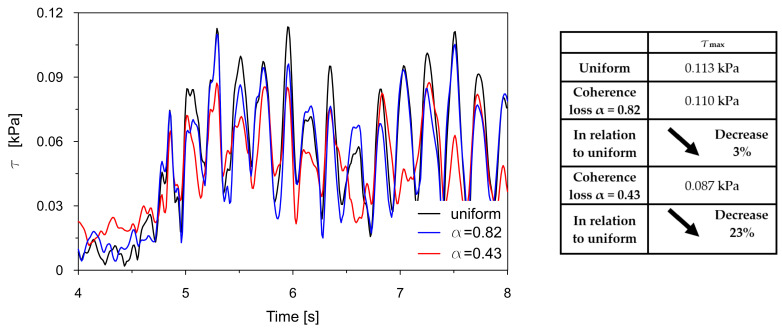
Comparison of the Tresca stresses at point P3, located close to the dam base in the clay core, for the uniform excitation (black line) and the non-uniform excitation with the approximate space scale parameter *α* = 0.82 (blue line) and the precise one *α* = 0.43 (red line).

**Figure 18:**
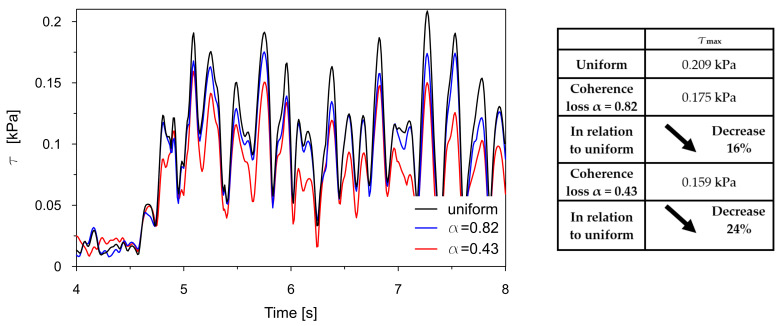
Comparison of the Tresca stresses at point P8, located in the middle of the coarse-grained dam body, for the uniform excitation (black line) and the non-uniform excitation with the approximate space scale parameter *α* = 0.82 (blue line) and the precise one *α* = 0.43 (red line).

**Figure 19:**
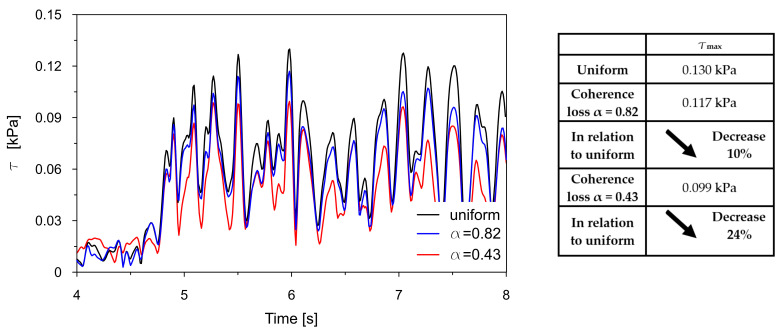
Comparison of the Tresca stresses at point P9, located close to the dam base in the clay core, for the uniform excitation (black line) and the non-uniform excitation with the approximate space scale parameter *α* = 0.82 (blue line) and the precise one *α* = 0.43 (red line).

The time traces (4–8 s) of Tresca stresses at the representative points P2, P3, P8, and P9 were compared for uniform excitation and for non-uniform excitation using an approximate space scale parameter (α = 0.82) as well as a precise one (α = 0.43). These comparisons are illustrated in Figure 16, Figure 17, Figure 18 and Figure 19.

The comparisons presented in Figure 16, Figure 17, Figure 18 and Figure 19 reveal a consistent trend across the representative points. The seismic response of the dam calculated under non-uniform kinematic excitation is consistently lower than that obtained under uniform excitation. Specifically, when using a space scale parameter of 0.82—an approximate value determined for the surrounding Carpathian flysch—the response is reduced by up to 16%. When applying the more accurate value of 0.43, derived specifically for the fractured foundation beneath the dam, the reduction reaches up to 24%.

This trend is consistent with the fact that the surrounding Carpathian flysch material is an unfractured, solid bedrock, where the decay of seismic wave coherence is lower than in the fractured bedrock material beneath the dam. As a result, the dam’s seismic response assuming a higher space scale parameter is closer to the response obtained under the assumption of uniform kinematic excitation.

The only exception is the trend observed at point P2, which is in the high-stiffness drainage zone directly adjacent to the bedrock. In this case, the level of seismic response is higher when the non-uniform kinematic excitation model is applied. With a space scale parameter of 0.82, an increase in stress of 14% is observed. This effect is attributed to changes in the foundation geometry during the seismic event and to the so-called quasi-static effects. A similar phenomenon has been reported by other researchers [21,22,24].

Generally, spatial variability in the seismic input, particularly coherence loss, significantly influences the stress distribution within the dam. The analysis confirmed that uniform excitation led to overestimated seismic responses compared to more realistic non-uniform inputs. Moreover, the appropriate value of the space scale parameter must be considered to avoid the overestimation of seismic demands. Preferably, this parameter should be determined experimentally, considering the geological and topographic characteristics of the bedrock material located directly beneath the dam. The use of experimental data obtained even in the close neighborhood of the dam may lead to a non-negligible underestimation or overestimation of the results.

### 3.3. Assessment of the Seismic Performance of the Dam Under SVEGM, Considering Coherence Loss and Wave Passage Effects

After establishing the space scale parameter of 0.43, appropriate for the fractured bedrock beneath the dam, the model of non-uniform kinematic excitation was enhanced to include the wave passage effect. The experimentally established wave velocity of 2800 m/s (see Section 3.1) was introduced to account for the shift in time delay.

**Figure 20:**
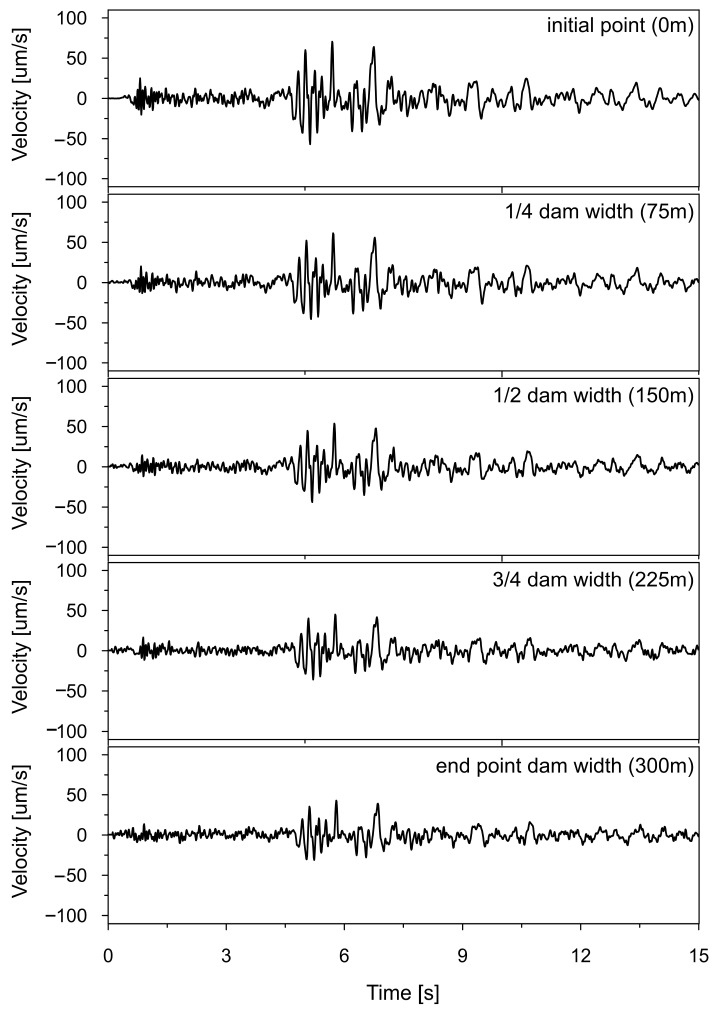
Input time histories of ground velocity in vertical (Z) direction of non-uniform kinematic excitation accounting for wave coherence loss (α = 0.43) and wave passage effect (v = 2800 m/s) at the beginning, 1/4, 1/2, 3/4, and the end of the dam foundation.

Time histories of the vertical ground velocity, resulting from non-uniform kinematic excitation with the space scale parameter α = 0.43 and wave passage velocity v = 2800 m/s, are shown in Figure 20. These were used as input at the beginning, 1/4, 1/2, 3/4, and end of the dam foundation.

**Figure 21:**
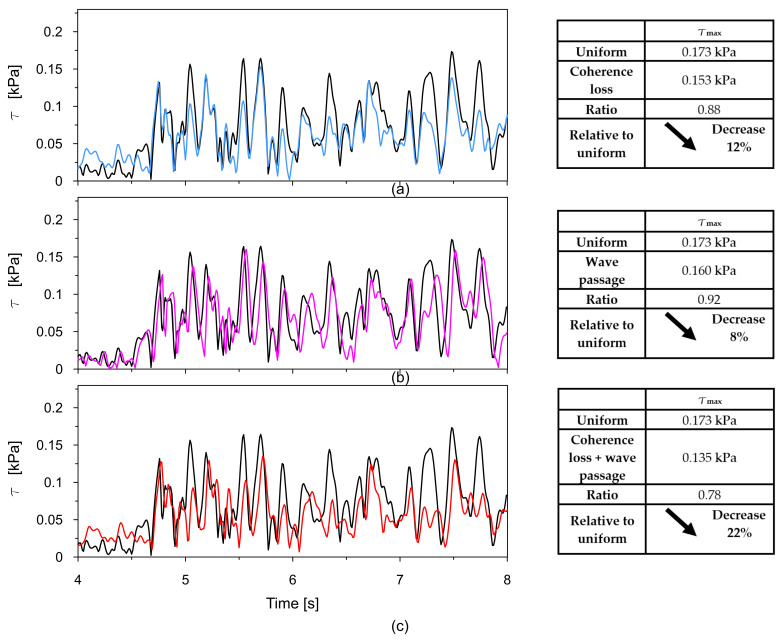
Comparison of Tresca stresses at point P2 obtained from the uniform excitation model with those from three non-uniform excitation scenarios: (**a**) model incorporating coherence loss using the experimentally determined space scale parameter α = 0.43; (**b**) model accounting for the wave passage effect with a wave velocity of 2800 m/s; and (**c**) model combining both coherence loss and the wave passage effect.

The time histories (4–8 s) of Tresca stresses at the representative point P2, obtained from the uniform excitation model, were compared with those from three non-uniform excitation scenarios: (a) the model incorporating the experimentally determined space scale parameter α = 0.43; (b) the model accounting for the wave passage effect with a wave velocity of 2800 m/s; and (c) the model combining both coherence loss and the wave passage effect. These comparisons are presented in Figure 21a, Figure 21b, and Figure 21c, respectively.

Based on the presented results comparing the maximum shear stresses under different models of excitation, several observations can be made regarding the influence of spatial variability effects on the seismic response of the dam.

In the case of uniform excitation, the maximum shear stress reaches 0.173 kPa. When coherence loss is introduced, the maximum stress decreases to 0.153 kPa, reflecting a 12% reduction. This indicates that incoherent wave propagation leads to a significant mitigation of the dam response, likely due to the averaging of asynchronous input signals.

When considering the wave passage effect, which accounts for the finite velocity of seismic wave travel and the resulting delay in excitation across a structure, the shear forces decrease to 0.160 kPa. This corresponds to an 8% reduction compared to uniform loading. Although less pronounced than the effect of coherence loss alone, wave passage still contributes to a noticeable decrease in dynamic stress, highlighting the importance of accounting for arrival time differences in spatially varying excitation.

The most substantial decrease in stress is observed when both coherence loss and the wave passage effect are considered simultaneously. In this combined scenario, a 22% reduction is observed relative to the uniform case. This suggests a synergistic interaction between the two phenomena, where the asynchronous excitation is further diffused by phase shifts introduced by the wave travel delay.

**Figure 22:**
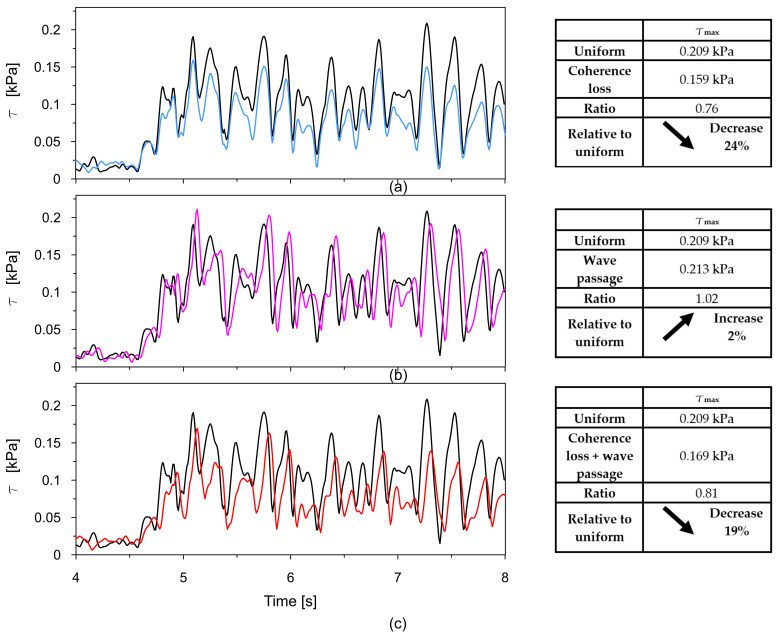
Comparison of Tresca stresses at point P8 obtained from the uniform excitation model with those from three non-uniform excitation scenarios: (**a**) model incorporating coherence loss using the experimentally determined space scale parameter α = 0.43; (**b**) model accounting for the wave passage effect with a wave velocity of 2800 m/s; and (**c**) model combining both coherence loss and the wave passage effect.

Similar to Figure 21, Tresca stress time histories at point P8 from the uniform excitation model were compared with the same three non-uniform scenarios, with the results presented in Figure 22a–c.

In this scenario, the influence of spatial variability effects on the maximum shear stress shows a slightly different pattern compared to the previous case.

Under uniform excitation, the maximum shear is 0.209 kPa. When coherence loss is introduced, this value decreases significantly to 0.159 kPa, indicating a 24% reduction. This confirms that the loss of coherence, which reflects the lack of synchronization in amplitude and phase between different excitation points, substantially diminishes the structural response by dispersing the input energy.

Interestingly, when the wave passage effect is considered on its own, the maximum stress slightly increases to 0.213 kPa—2% higher than under uniform excitation. This result differs from the earlier scenario and suggests that in certain configurations, the delay in wave arrival across the dam led to an amplification of the dynamic response.

When both coherence loss and the wave passage effect are applied simultaneously, the maximum stress is 0.169 kPa, which is a 19% decrease compared to the uniform excitation case. This combined reduction is smaller than the one caused by coherence loss alone, indicating that the wave passage effect may, in this scenario, partly counteract the stress-mitigating influence of incoherent excitation.

The stress levels in the dam resulting from the shock remain well below the permissible limits for the earth structure, as the peak ground velocity values of this shake are far below the maximum levels predicted for this region. In the case of an earthquake reaching the maximum predicted values for this area, the dynamic response of the dam could be significantly greater.

## 4. Conclusions

The results obtained allow the formulation of some important conclusions concerning the seismic performance of the earth dam:

1. Bedrock material properties significantly influence the seismic response of earth dams. This study confirmed that fractured bedrock beneath the dam leads to lower seismic wave velocities and a reduced space scale parameter compared to undisturbed surrounding formations of the Carpathian flysch.
2. A space scale parameter must reflect actual foundation conditions. Using a parameter determined for surrounding intact rocks (e.g., Carpathian flysch) instead of the fractured bedrock beneath the dam can lead to errors in stress prediction.
3. Uniform excitation models overestimate the dynamic response of dams. The comparison showed that using a uniform seismic input and ignoring spatial variability can result in an overestimation of shear stresses by up to 24%.
4. The spatial variability in ground motion mitigates the seismic response of the dam. However, local effects may override global trends. At some points—especially near interfaces with stiff layers or drainage zones—non-uniform excitation resulted in increased stresses. This can originate from quasi-static effects, resulting from changes in the bedrock’s geometry during the seismic event.
5. The combined modeling of coherence loss and the wave passage effect produces the most realistic results. When both effects were included, the predicted stresses were significantly lower than in uniform models—by up to 22% in some zones. This indicates a synergistic effect that should not be neglected.

This study demonstrates that site-specific bedrock material conditions significantly affect the seismic behavior of earth dams, highlighting the importance of incorporating appropriate spatiotemporal parameters of bedrock in structural assessments. Accurately capturing local geological variations leads to more reliable evaluations of seismic safety and stress distribution in large-scale dams. Therefore, future research will focus on further field experiments to directly measure wave passage and incoherence effects in various bedrock types, allowing for the refinement of the spatiotemporal input parameters. Moreover, the analysis will be extended to include different dam geometries, material usage, and construction types of dams to assess the broader applicability of the results and the overall validity of the findings.

## Figures and Tables

**Table 1:** Mechanical properties obtained from field studies of the dam’s bedrock formations [38,39], as well as shear and compressional wave velocities.

Rock Formation	Elastic Modulus *E* [GPa]	Poisson’s Ratio *ν* [–]	Shear Wave Velocity *v_s_* [m/s]	Compressional Wave Velocity *v_p_* [m/s]
Limestones	20–35	0.30–0.37	2420	5550
Marly limestones	20	0.36	1790	3830
Marls	11.5	0.36	1360	2910
Sandstones	15–21	0.38	1820	4140
Radiolarites	12–20	0.33–0.37	1810	3590
Siliceous limestones	13–14.5	0.32–0.38	1550	3020
Hornstones and shales	14–15	0.32–0.38	1570	3050

**Table 2:** Elastic moduli adopted in the particular zones of the numerical model of the dam.

	Elastic Modulus [MPa] in the Zone:				
	1	2	3	4	5
Dam body (air side)	495	670	830	960	1016
Dam body (water side)	414	722	894	1023	1120
Protective sand layer, 1 m thick (air side)	213	335	462	577	640
Protective sand layer, 1 m thick (water side)	215	389	507	566	610
Protective gravel layer, 3 m thick (air side)	510	680	875	1024	1098
Protective gravel layer, 3 m thick (water side)	637	830	984	1041	1199
Dam core	110	190	228	269	295
Drainage layer	830	984	1148	1230	1280

**Table 3:** The main parameters of the Podhale seismic shock on 2 December 2004.

Parameter	The 2 December 2004 Podhale Shock
Epicenter location	Czarny Dunajec
Magnitude	3.6
Duration	30 s
Strong-intensity phase	15 s
Shock energy	1.58 × 10^10^ J
PGV Station 1, WE direction	89.74 μm/s
PGV Station 1, NS direction	89.72 μm/s
PGV Station 1, Z direction	70.54 μm/s
PGV Station 2, Z direction	76.11 μm/s
PGV Station 3, Z direction	66.98 μm/s
Dominant frequency range	1–2 and 4–5 Hz with a peak at 2.3 Hz

## Data Availability

The original contributions presented in this study are included in the article. Further inquiries can be directed to the corresponding author.
